# Fully automated virtual screening pipeline of FDA-approved drugs using Caver Web

**DOI:** 10.1016/j.csbj.2022.11.031

**Published:** 2022-11-17

**Authors:** Milos Musil, Andrej Jezik, Marie Jankujova, Jan Stourac, Jakub Galgonek, Saltuk Mustafa Eyrilmez, Jiri Vondrasek, Jiri Damborsky, David Bednar

**Affiliations:** a Loschmidt Laboratories, Department of Experimental Biology and RECETOX, Masaryk University, Brno, Czech Republic; b International Clinical Research Center, St. Anne's University Hospital Brno, Brno, Czech Republic; c Faculty of Information Technology, Brno University of Technology, Brno, Czech Republic; d Institute of Organic Chemistry and Biochemistry of the CAS, Prague, Czech Republic

**Keywords:** Caver, CaverDock, FDA-approved drug, Channel, Tunnel, Virtual screening, Web, FDA, U.S. Food and Drug Administration, IDSM, Integrated Database of Small Molecules, PDB, Protein Data Bank, CIF, Crystallographic Information File, CSA, Catalytic Site Atlas

## Abstract

Protein tunnels are essential in transporting small molecules into the active sites of enzymes. Tunnels' geometrical and physico-chemical properties influence the transport process. The tunnels are attractive hot spots for protein engineering and drug development. However, studying the ligand binding and unbinding using experimental techniques is challenging, while *in silico* methods come with their limitations, especially in the case of resource-demanding virtual screening pipelines. Caver Web 1.2 is a new version of the web server combining the capabilities for the detection of protein tunnels with the calculation of the ligand trajectories. The new version of the Caver Web server was expanded with the ability to fetch novel ligands from the Integrated Database of Small Molecules and with the fully automated virtual screening pipeline allowing for the fast evaluation of the predefined set of over 4,300 currently approved drugs. The virtual screening pipeline is accompanied by a comprehensive user interface, making it a viable service for the broader spectrum of companies and the academic user community. The web server is freely available for academic use at https://loschmidt.chemi.muni.cz/caverweb.

## Graphical abstract

**Figure:**
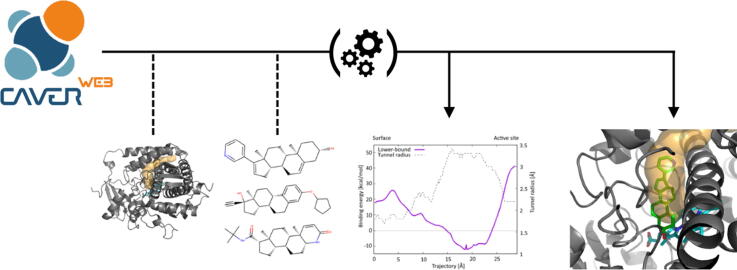

## 1 Introduction

The search for new chemical compounds and their application in therapeutic areas is in the midst of the interest of the pharmaceutical industry. In recent years, a phenotype-based discovery has come at the peak of interest of various research groups [1], [2]. However, the continuous efforts in solving protein structures and the wide availability of the modelling tools such as AlphaFold2 [3] made the target-based method a staple procedure in drug discovery. The majority of drugs were obtained using this method [4].

The human genome project has unearthed approximately 20,000 protein-encoding genes [5], [6] producing over 10,000 ubiquitously expressed proteins [7], [8]. However, less than ten percent of those proteins are currently targeted by the approved drugs [9] leaving a vast number of potential therapeutic targets that could be potentially exploited. Developing new drugs and their delivery to the market is expensive and laborious. It has been estimated that from the initial inception to the market distribution, the cost of a new drug can go over 2 billion US dollars [10], [11]. Therefore, repurposing approved drugs seems a viable strategy to reduce the costs and time demands for novel drug identification.

High-throughput robotics screenings are still time-demanding and require expensive infrastructure. On the other hand, with the recent advances in the fields of information technology and computational biology, *in silico* techniques provide an exciting alternative to experimental screening [12], [13], [14], [15]. Those methods can quickly evaluate vast libraries from which only a tiny fraction of the top binders are typically considered for further experimental confirmation [16]. Therefore, virtual screening methods have become a prominent technology in the probing and prioritization of drug-like compounds in the pharmaceutical industry [17]. The vast majority of virtual screening platforms score the binding of inhibitors to the binding or active-site pockets. However, the access of ligands to these functionally important sites is rarely considered.

Our *in-house* web tool Caver Web [18] incorporated both Caver [19], software for identification and geometrical analysis of tunnels, and CaverDock [12], software for docking-based analysis of the ligand transport. Therefore, even inexperienced users with limited bioinformatics knowledge can efficiently study the ligand transport process. However, in the current implementation, only a single ligand could be analyzed in each run of Caver Web. This was a limiting step for the users looking to identify the best-fitting inhibitors for their protein of interest.

Here, we present a new version of Caver Web that overcomes those shortcomings, making it a viable service for the scientific community and the broader spectrum of companies. A new version of the Caver Web server provides a fully automated virtual screening pipeline using the predefined set of the medical drugs approved by major world health organizations (FDA+) that contains over 4,300 ligands obtained from the ZINC database [20]. Furthermore, Caver Web was extended with the possibility of fetching the ligands directly from the Integrated Database of Small Molecules (IDSM) [21]. Finally, the graphical user interface of the Caver Web service was expanded with new elements for the visualization of the transition energies and the bottleneck detection based on the transition of the ligands through tunnels. The server is freely available for the scientific community at https://loschmidt.chemi.muni.cz/caverweb.

## 2 Materials and methods

**Fig. 1:**
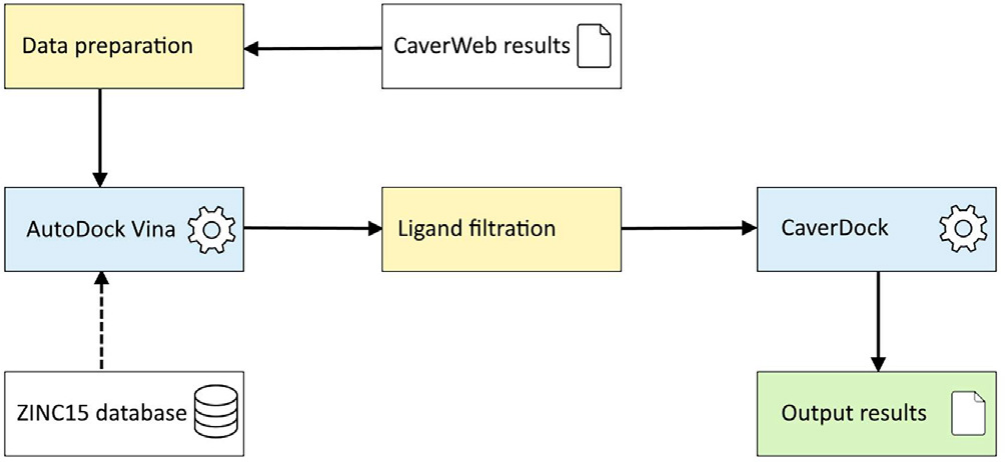
Simplified workflow of the virtual screening pipeline. The calculation is done in two critical steps: (i) evaluating all FDA+ drugs using the Autodock Vina and (ii) the assessment of the best hits using the CaverDock calculation.

The basic workflow of the implemented virtual screening pipeline is captured in Fig. 1. The virtual screening can be started only after the successful run of the Caver Web calculation, which requires only the protein structure in the PDB or CIF format. The identification of pockets using the Fpocket 2 [22], the search for the essential residues in the mCSA [23] and SwissProt [24], and the calculation of the tunnels employing Caver 3.02 [19] is processed during the standard run of the Caver Web service.

### 2.1 Virtual screening of the FDA-approved drugs

Several preparation steps must be initiated to proceed with the fully automated virtual screening pipeline. Once the user selects the tunnel in the Caver Web interface and the tunnel data are obtained from the server, the data preparation is initiated. In this step, the prepare_receptor4.py script from the MGLTools package [25] is utilized to add Gasteiger charges and AutoDock Vina [15] compatible atom types to every atom in the protein structure. A tunnel discretizer [26] is then employed to cut the tunnel into discrete slices with specified distances. Finally, the predefined set of the approved drugs (4,381 ligands from the ZINC database) is prepared using the MGLTools prepare_ligand4.py script. The discretized tunnel, receptor, and the collection of ligands are then forwarded to be processed by the Autodock Vina and in-house tool CaverDock [12].

Due to the large input library size, it is impossible to evaluate all of the world-wide-approved drugs with the comprehensive and time-demanding CaverDock calculation. Therefore, in the first step, the binding energy of each ligand in the protein's active site is rapidly evaluated by the fast AutoDock Vina algorithm. Ligands in the library are then sorted based on their predicted binding energy, and the top 50 binders are evaluated using the CaverDock algorithm. Users can modify the discretization parameter defining the distance between the centres of two slices of the tunnel and select the residues that should be considered for calculation. Both the bottleneck energy of the lower-bound trajectory and the lowest energy of the bound ligand are provided to the user. However, the binding energy is always set as a default quality metric when sorting the ligands calculated by CaverDock.

### 2.2 Integrated database of small molecules

The current implementation of the Caver Web service allowed users to start their ligand transport analysis using either PDBQT/MOL, SMILES, or ZINC code identifiers. However, there was no possibility of searching for the ligands of interest in the existing databases. For this reason, we have expanded our server with the IDSM service [21] that combines data from several sources, mainly PubChem [27], ChEMBL [28], and ChEBI [29] small-molecule datasets. It is now possible to fetch ligands directly from the IDSM database using either the ligand's approximate name or its SMILES code. Furthermore, we have implemented a more robust sorting algorithm to obtain the most relevant results on top of the list. As a result, the integration of the IDSM database enables users to start their ligand transport analysis without the need to search for the PDBQT/MOL representation or SMILES codes first.

### 2.3 Graphical user interface

**Fig. 2:**
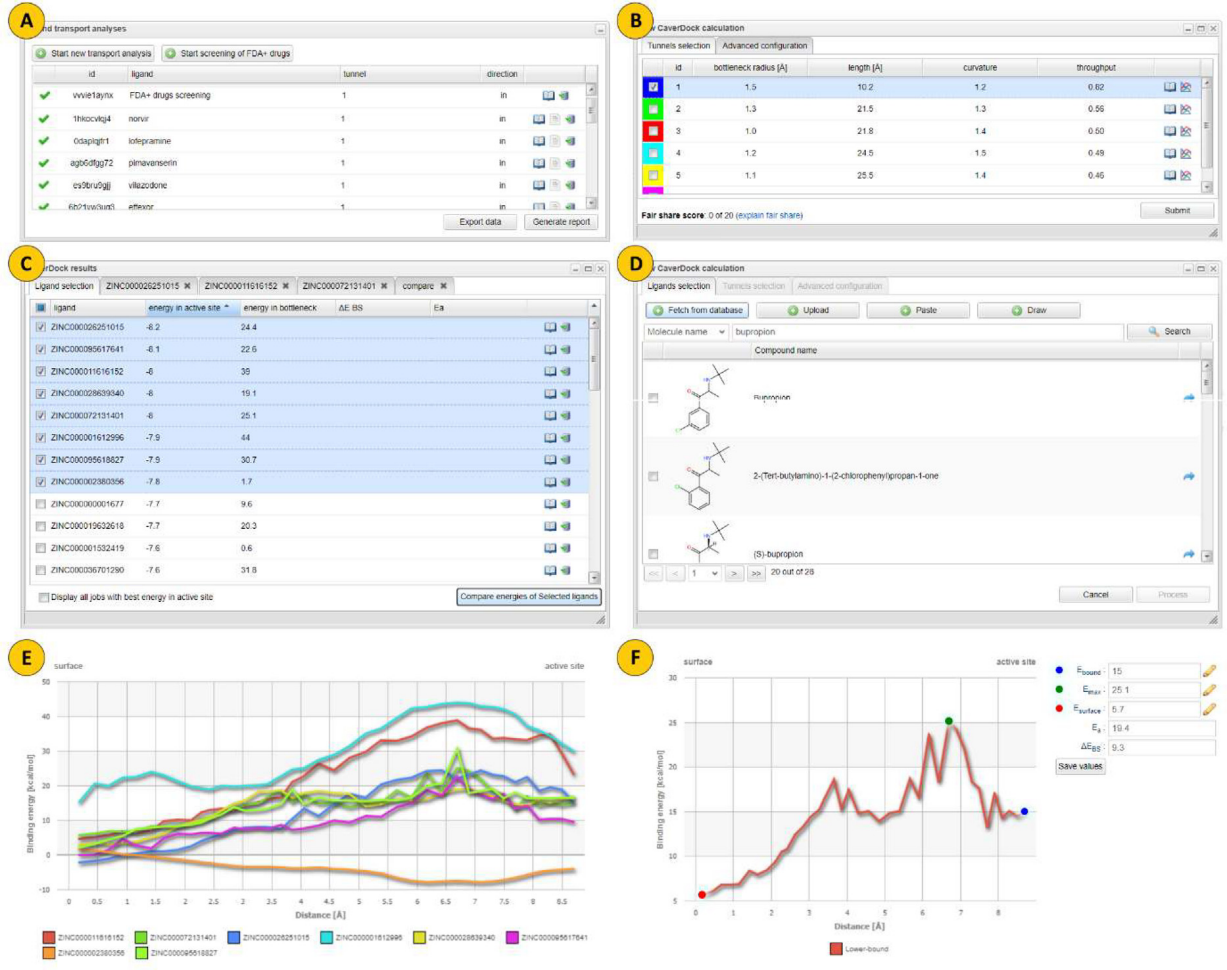
Graphical user interface of the virtual screening pipeline. A) Ligand transport analysis panel allows users to analyze either a specific ligand or start the virtual screening pipeline using the predefined set of FDA+ drugs. B) Tunnel selection/settings for the virtual screening pipeline. C) The Results table shows the best-fitting ligands. D) Selection of the specific ligand for the CaverDock calculation using the IDSM database. E) Visualization of the calculated binding energies for the whole trajectory. F) Comparison of the energy profiles of the selected ligands.

The interactive user interface for the virtual screening pipeline was implemented as a natural extension of the original Caver Web server. The transport analysis option was expanded with the possibility of fetching ligands directly from the IDSM database (Fig. 2**D**). The virtual screening of the FDA+ drugs on the target protein can be started by simply clicking the “Start screening of the FDA+ drugs” button (Fig. 2**A**). Here, selecting the tunnel of interest is possible, together with the setting of the discretization radius and the selection of the residues that should be kept for the calculation (Fig. 2**B**). Once the virtual screening is finished, it is possible to download raw data as an Excel sheet or generate a full report in PDF. The PDF report contains a plot of lower-bound trajectories for each evaluated ligand and the dot-plot showing the energy minima of all ligands based on their position in the protein tunnel.

By clicking on the book icon, the table with the 50 most promising ligands can also be directly visualized in the web interface (Fig. 2**C**). The table contains the binding energy of the selected ligands in the protein's active site and in the bottleneck of the analyzed tunnel. Furthermore, by clicking on the book icon, it is possible to open a new tab for any of these ligands to visualize the plot of the calculated binding energies for the whole trajectory. This window allows the user to define points for calculating the activation energy of protein–ligand association and the energy difference between ligand bound on the surface and in the active site (Fig. 2**E**). The energy profiles of several ligands can be directly compared in a single plot (Fig. 2**F**). Finally, the ligand transport from the surface to the protein’s active site is generated as a PyMOL session [30] for each of the best-performing ligands. It is available via *Download results in a single zip* button.

## 3 Results

We have evaluated our virtual screening pipeline using the Cytochrome P450 17A1 (CYP17A1). It is one of the most crucial enzymes in the steroidogenic pathway that produces glucocorticoids, androgens, progestins, and estrogens. In humans, CYP17A1 is associated with endocrine effects and steroid hormone metabolism. The mutations in the CYP17A1 gene are therefore connected with the pseudohermaphroditism [31], [32], [33], and adrenal hyperplasia [34], [35]. Furthermore, the decreased enzyme activity is related to infertility due to hypogonadotropic hypogonadism [36]. CYP17A1 is also an essential target in the treatment of prostate cancer as its inhibition leads to the lower production of androgen required for tumour cell growth [37], [38], [39], [40]. The enzyme is expressed by most steroidogenic tissues, including testes, ovaries, and the adrenal cortex. However, it has also been detected in the heart, kidney, and adipose tissue [41].

CYP17A1 crystal structure (PDB ID: 3RUK [42]) was used as an input for the Caver Web calculations. As the enzyme contains an active site that associates with a heme prosthetic group [43], the heme cofactor was selected as a starting point for detecting the active site necessary for tunnel calculation. The integrated Fpocket2 software [22] also confirmed the same region. Heme cofactor was set to be preserved in the structure, and the remaining settings were kept in their default values.

Caver Web detected 17 tunnels in total, with only four tunnels being opened more than 1 Å along the whole tunnel. From the list of suggested tunnels, the most biologically relevant tunnel [44] No. 2 was selected for the virtual screening using the predefined set of FDA+ drugs, and the results were compared with the known inhibitors of CYP17A1. We have also evaluated the remaining three well-opened tunnels. However, those tunnels are only modifications of the main tunnel with a smaller opening on the protein’s surface or show a significantly higher energetic barrier during the ligand transport.

Abiraterone is the only FDA-approved drug used to treat castration-resistant prostate cancer by inhibiting the CYP17A1, where it binds in its active site and permanently disables the enzyme [42], [45]. Abiraterone replaced now rarely applied Ketoconazole which inhibits CYP17A1 competitively, making its effectiveness dependent on the concentration of the administered drug [46]. Two novel drugs, Galeterone and Seviteronel, were investigated as potential inhibitors of CYP17A1. However, those drugs are not included in the set of 4,381 FDA+ drugs as the development of Galeterone was discontinued in 2017.

**Fig. 3:**
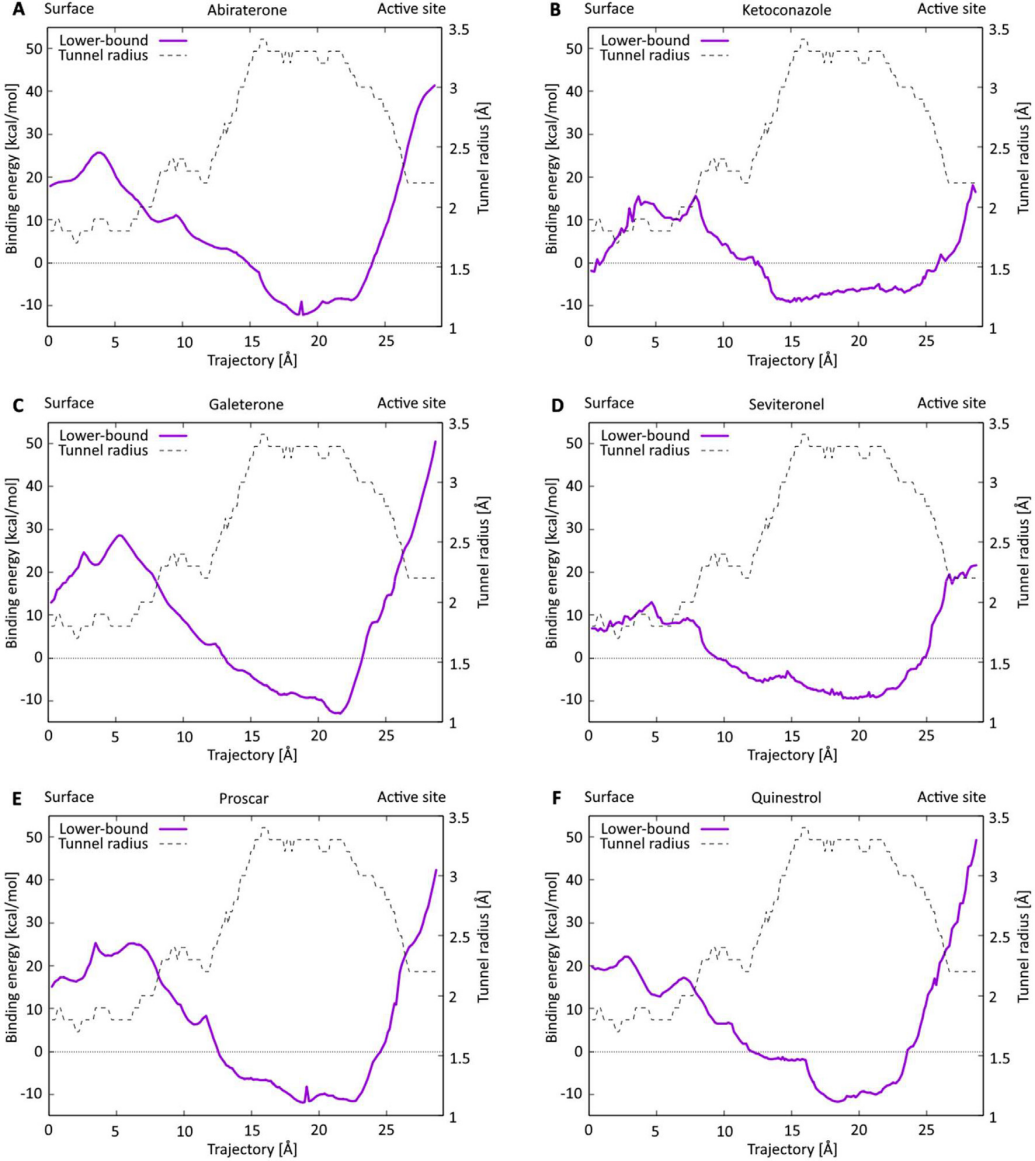
Lower-bound energies of the ligand transport among the tunnel geometry. The list contains FDA-recognized inhibitors of CYP17A1 Abiraterone (**A**) and Ketoconazole (**B**), currently investigated novel inhibitors Galeterone (**C**) and Seviteronel (**D**), and the best-scoring ligands from the fully automatized scan of the FDA+ drugs Proscar (**E**) and Quinestrol (**F**).

Firstly, we individually evaluated all four previously described inhibitors by searching them in the IDSM database [21]. The results of the ligand transport analysis can be observed in Fig. 3**A-D**. We can observe that after passing the bottleneck residue Asn202 responsible for the inhibitor’s selectivity in the CYP17A1 enzyme [44], [47], the Abiraterone binds in the active site of the enzyme with a low binding energy of −11 kcal/mol. A similar result was achieved with Galeterone showing a slightly higher energetic barrier around the gateway residue and lower binding energy of −13 kcal/mol in the active site of the enzyme. The older Ketoconazole shows a lower energetic barrier and a slightly higher binding energy of about −9 kcal/mol. Furthermore, the lowest binding energy of Ketoconazole is reached about 4 and 7 Å closer to the protein surface compared to Abiraterone and Galeterone, respectively. This would suggest that Ketoconazole does not bind directly in the active site and coordinate the heme iron such as Abiraterone through its pyridine nitrogen [42]. Finally, the novel Seviteronel does not show as promising binding energies as its Galeterone counterpart. However, its energetic barrier is about half of that of Abiraterone. This would make it easier for the ligand to reach the enzyme‘s active site but also to leave it.

Secondly, we conducted a fully automated scan of the entire FDA+ database to analyze the positions of the known inhibitors in the list of all 4,381 FDA+ drugs. Since the development of Galeterone was discontinued, and Seviteronel is not yet included in the world-wide-approved set of the ZINC database, the analysis was limited to Abiraterone and Ketoconazole. Four variants of Abiraterone are located in the FDA+ list of ligands in the ZINC database. All four were among the top hits of our fully automated scan, with the Abiraterone acetate sold under the brand name Zytiga scoring 15th place from the whole set of 4,381 ligands. Ketoconazole was not placed among the fifty best-scoring ligands with a binding energy of 2 kcal/mol lower than Abiraterone. Interestingly, the first two positions on the list were occupied by Proscar (Fig. 3**E**) and Quinestrol (Fig. 3**F**). Proscar is a 5α-Reductase inhibitor that has (similarly to Abiraterone) an antiandrogenic effect and is currently used for the treatment of enlarged prostate. It has been shown to decrease the risk of low-grade prostate cancer. However, the risk of high-grade prostate cancer may increase due to the treatment of some early symptoms [48], [49]. Quinestrol is a synthetic estrane steroid already employed as a prostate cancer medication [50]. The top hits list contained several more drugs with a similar purpose (Avodart, Estradiol Benzoate), while the vast majority of the suggested drugs were related to the steroidogenic pathway. Thus, our computational analysis has revealed a large set of drugs that could possibly be repurposed to complement the Abiraterone used as prostate cancer medication. The robustness of our approach could be further substantiated by the fact that Galeterone would be placed on the top of the list if it was already contained in the list of FDA+ drugs.

**Fig. 4:**
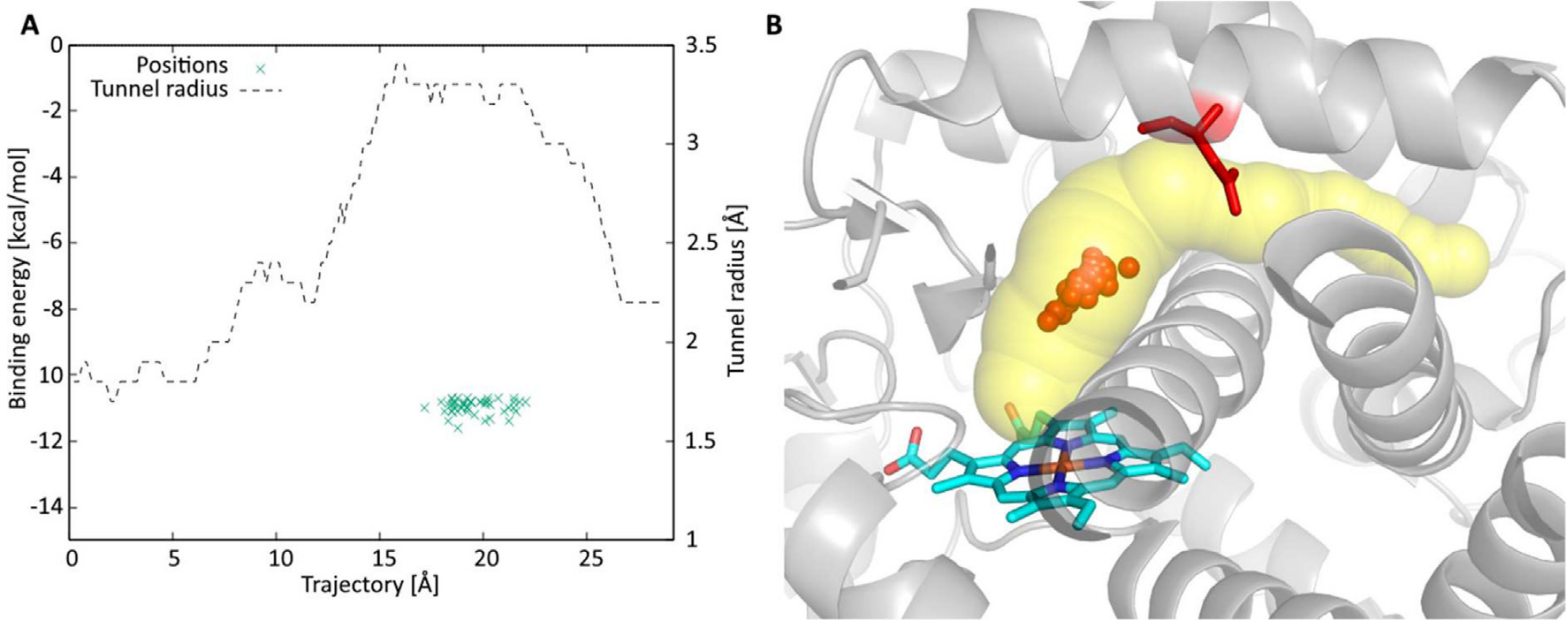
(**A**) Position of the lowest binding energies in the proteins tunnel (green crosses) for the set of fifty best-scoring ligands from the fully automatized scan of the FDA+ drugs. (**B**) The visualization of the enzyme tunnels with the bound best-scoring ligands. The heme cofactor is coloured in cyan; the bottleneck residue Asn202 is highlighted in red. Red spheres denote the positions of bound ligands in the enzyme tunnel. Their locations were calculated as centres of mass of the bound ligands. (For interpretation of the references to colour in this figure legend, the reader is referred to the web version of this article.)

Finally, Fig. 4 captures the positions of the lowest binding energies of the fifty best-scoring ligands in the enzyme tunnel. From here, it can be observed that all of the suggested ligands are bound directly in the active site of the enzyme, thus interacting with its heme prosthetic group. The ligand transport of the best-scoring drugs can be further analyzed in the Supplementary material (PyMOL session, MPEGs of the ligands transport, Excel sheet, and raw data in CSV). The data provided in the supplementary files show almost negligible differences in the top-scoring ligands as the binding energies of the first and the last of the suggested ligands differ in less than 1 kcal/mol. In general, 0.5 kcal/mol deviation can be attributed to the heuristic nature of algorithms, and therefore, the order of ligands can slightly change in each consecutive run. Thus, the user should not consider only one top-scoring molecule as the final inhibitor but rather select several hits for experimental validation.

## 4 Conclusions

The development of new sequencing strategies and the large genome projects have unearthed the existence of thousands of ubiquitously expressed human proteins from which currently approved drugs target only a tiny fraction. This leaves many proteins to be further exploited by pharmaceutical research. *In silico *techniques provide a fast and cheap alternative to high-throughput screening. However, they are hard to set up, especially when dealing with the binding sites buried inside the protein structure.

Caver Web is a web tool that incorporates Caver and CaverDock software to provide users with a one-stop-shop solution for the identification and geometrical analysis of tunnels and the calculation of the energy profile of the ligand transport along the selected tunnels. Both aforementioned tools were previously successfully utilized in various studies, including spike glycoprotein of SARS-CoV-2 [51], and leukotriene [44]. However, in the previous version of Caver Web, CaverDock was limited only to a single ligand analysis. A fully automated virtual screening pipeline using the predefined set of 4,381 FDA+ drugs obtained from the ZINC15 database was newly implemented into the Caver Web interface. Users can start the scan of the FDA+ drugs with just a few clicks, and the results are generated automatically. The energy profile of the ligand transport can be visualized for each ligand or compared to other ligands in a single plot. The transport path is generated in the form of a PyMOL session, and all the results can be downloaded as an Excel sheet or PDF report. Furthermore, for the transport analysis of the individual ligands, the IDSM database was integrated to allow the users to fetch ligands outside the ZINC database's scope easily.

Our virtual screening pipeline was evaluated on the cytochrome P450 17A1, and the results were compared with the known CYP17A1 inhibitors. We have analyzed the transport path of Abiraterone, Ketoconazole, Galeterone, and Seviteronel. According to our expectations, Abiraterone has shown superior binding energies compared to the older Ketoconazole. Furthermore, previously investigated Galeterone slightly surpasses Abiraterone binding capabilities (approximately 2 kcal/mol), while Seviteronel offers a significantly lower energetic barrier (approximately 13 kcal/mol) around the enzyme's bottleneck residue. In the second step, we run a fully automated virtual screening pipeline using the entire set of 4,381 FDA+ drugs. All variations of Abiraterone contained in the ZINC database have scored among the best potential inhibitors for the CYP17A1 enzyme with a negligible difference in binding energies of less than 1 kcal/mol compared to the best-scoring ligand. In contrast, many best-scoring drugs show the same or similar function to Abiraterone. This leaves us with a large set of drugs that could be potentially repurposed as Abiraterone alternatives.

To conclude, the new version of Caver Web introduces a fast and effective virtual screening pipeline combining molecular docking with ligand transport analysis over a wide range of ligands, thus making it a viable service for the broader spectrum of the scientific community. The server is freely available for non-commercial use at https://loschmidt.chemi.muni.cz/caverweb.

## CRediT authorship contribution statement

**Milos Musil:** Conceptualization, Supervision, Software, Formal analysis, Writing – original draft, Writing – review & editing. **Andrej Jezik:** Software, Methodology. **Marie Jankujova:** Software, Visualization. **Jan Stourac:** Conceptualization, Software. **Jakub Galgonek:** Software, Writing – review & editing. **Saltuk Mustafa Eyrilmez:** Validation. **Jiri Vondrasek:** Conceptualization, Supervision. **Jiri Damborsky:** Conceptualization, Supervision, Writing – review & editing, Funding acquisition. **David Bednar:** Conceptualization, Supervision, Writing – review & editing, Project administration, Funding acquisition.

## Declaration of Competing Interest

The authors declare that they have no known competing financial interests or personal relationships that could have appeared to influence the work reported in this paper.
